# Effect of Intraoperative Corneal Stromal Pocket Irrigation in Small Incision Lenticule Extraction

**DOI:** 10.1155/2015/928608

**Published:** 2015-07-27

**Authors:** Yu-Chi Liu, Lasitha Jayasinghe, Heng Pei Ang, Nyein Chan Lwin, Gary Hin Fai Yam, Jodhbir S. Mehta

**Affiliations:** ^1^Singapore Eye Research Institute, Singapore; ^2^Singapore National Eye Centre, 11 Third Hospital Avenue, Singapore 168751; ^3^Department of Clinical Sciences, Duke-NUS Graduate Medical School, Singapore; ^4^School of Material Science & Engineering and School of Mechanical and Aerospace Engineering, Nanyang Technological University, Singapore

## Abstract

This study aimed at evaluating the effect of intraoperative corneal pocket irrigation in small incision lenticule extraction (SMILE) and compares it to that in femtosecond laser-assisted in situ keratomileusis (FS-LASIK). Sixteen rabbit eyes underwent a SMILE procedure, with 8 eyes having corneal pocket irrigation, while the other 8 eyes were without irrigation. Another 16 eyes underwent a FS-LASIK procedure for comparison, with 8 eyes having flap irrigation, while the other 8 eyes were without irrigation. The results showed that the changes in the total corneal thickness, anterior and posterior lamellar thickness, measured by the anterior segment optical coherence tomography, were comparable between the SMILE with and without irrigation groups, suggesting that the irrigation did not lead to significant changes in the corneal thickness. However, at postoperative 8 hours, in vivo confocal microscopy showed that the interface reflectivity in the SMILE with irrigation group was significantly higher than that in other three groups. The presence of interface fluid was further confirmed by the identification of fluid pockets with undulated collagen shown on histological section in the post-SMILE with irrigation eyes. Our findings might contribute to the occurrence of post-SMILE delayed immediate visual quality recovery and further clinical study is required.

## 1. Introduction

The use of femtosecond laser technology has been shown to increase the safety, efficiency, precision, and versatility in refractive surgery [1]. Recently, small incision lenticule extraction (SMILE), a variation of refractive lenticule extraction (ReLEx) procedure, has become clinically available in Europe and Asia as an alternative to laser-assisted in situ keratomileusis (LASIK) for the correction of myopia [2]. In SMILE, a femtosecond laser is used to create an intrastromal lenticule with a small peripheral arcuate incision. Following lenticule creation, removal of the lenticule is performed by blunt separation of the anterior surface of the lenticule followed by the posterior surface [2]. The lenticule is then grasped and removed with forceps directly via the small incision. Following lenticule removal, most surgeons, including the authors, flush the intrastromal pocket with balanced salt solution (BSS) or saline [2–6]. This irrigation step is thought by surgeons to minimize infectious and noninfectious contaminants entering the pocket, to wash out the inciting inflammatory cytokines resulting from the surgical manipulations and laser lenticule creation, and to minimize the epithelial ingrowth related to epithelial cells introduced unintentionally by the surgeon through the small incision.

With regard to the visual and refractive outcomes, it has been shown that the efficacy, safety, and predictability profiles of SMILE for myopia or myopic astigmatism are good [2, 7–9] and patient satisfaction is high [6]. However, SMILE has its own challenges. Firstly, it is more technically demanding than LASIK and is with a steeper learning curve [10]. Secondly, it is more challenging when performing a low refractive correction, as handling a thin lenticule is more difficult [11]. Thirdly, a slight delay in the uncorrected distance visual acuity recovery in the early postoperative period has been reported after SMILE [8, 12], and the reasons for this are not fully elucidated. In a prospective clinical study conducted by our group, we have also reported that subjective symptoms of visual fluctuations and episodes of blurring of vision were greater after SMILE than LASIK in the early postoperative period [13]. This might result from (i) suboptimal laser cutting of the stromal fibres that subsequently causes interface scattering [12, 14] and (ii) microdistortions in Bowman's layer, revealed in anterior segment optical coherence tomography (ASOCT) images in post-SMILE patients [15]. Beside these possible causes, we wanted to assess the contribution of fluid used for irrigation in the SMILE procedure. We hypothesized that the irrigating fluid may be trapped in the corneal pocket, affecting the immediate postoperative visual quality. As opposed to SMILE, the fluid used for interface irrigation in FS-LASIK procedure is less likely to be retained at the interface due to the larger circumferential flap incision. Hence, we used a previous described rabbit model of SMILE and FS-LASIK [16, 17] to evaluate the effect of irrigation of the pocket in the SMILE procedure.

## 2. Methods

### 2.1. Study Animals and Experimental Groups

Sixteen 12-to-15-week-old New Zealand white rabbits with 3-4 kg body weight were obtained from National University of Singapore and housed under standard laboratory conditions. Thirty-two eyes were randomly allocated to four groups: femtosecond- (FS-) LASIK with irrigation, FS-LASIK without irrigation, SMILE with irrigation, and SMILE without irrigation groups (*n* = 8 per group). All animals were treated according to the guidelines of the Association for Research in Vision and Ophthalmology Statement for the Use of Animals in Ophthalmic and Vision Research. The protocol was approved by the Institutional Animal Care and Use Committee of SingHealth. All surgeries and evaluations were performed under general anesthesia with xylazine hydrochloride (5 mg/kg intramuscularly; Troy Laboratories, Smithfield, Australia) and ketamine hydrochloride (50 mg/kg intramuscularly; Parnell Laboratories, Alexandria, Australia). All the procedures were performed by an experienced refractive surgeon (Jodhbir S. Mehta).

### 2.2. Femtosecond-LASIK Procedure

Eight rabbits (*n* = 8 eyes) underwent a FS-LASIK procedure. Our rabbit experimental model for FS-LASIK was used as previously described [16]. LASIK flaps were created by using a 500 kHz femtosecond laser (VisuMax; Carl Zeiss Meditec, Jena, Germany). The laser parameters were as follows: 110 *μ*m flap thickness, 7.9 mm flap diameter, 170 nJ power, spot distance and tracking spacing of 4.8 *μ*m for lamellar flap and 2 *μ*m for flap side cuts, respectively, flap side cut at 90°, hinge position at 90°, hinge angle of 50°, and spiral in (centripetal) scanning pattern direction. After the flap was lifted, the underlying stroma underwent a 6.5 mm optical zone myopic ablation of −6.00 D using an excimer laser (Technolas; Bausch & Lomb, Rochester, NY) with the following laser parameters: spot size of 2.0 mm diameter, fluence of 120 mJ/cm^2^, and repetition rate of 100 Hz. Before the flap was repositioned, the stromal interface was gently irrigated with 1.5 mL BSS using a 25-gauge Buratto's cannula for the FS-LASIK with irrigation group, while the irrigation was not done for the FS-LASIK without irrigation group. Immediately after the procedure, the eyes received topical tobramycin ointment once (Alcon, Fort Worth, TX, USA).

### 2.3. Small Incision Lenticule Extraction (SMILE) Procedure

Eight rabbits (*n* = 16 eyes) underwent a SMILE procedure and were randomly divided to two groups: SMILE with BSS irrigation and SMILE without BSS irrigation groups (*n* = 8 eyes per group). Our rabbit experimental model for SMILE was performed as previously described [17]. A myopic correction of −6.00 D was performed with a 500 kHz femtosecond laser (Visumax; Carl Zeiss Meditec, Germany). The eye was docked on a small curved interface suction cone. The femtosecond incisions were performed in a spiral in/out scanning pattern direction [17]. The laser parameters were 120 *μ*m cap thickness, 7.5 mm cap diameter, and 6.5 mm lenticule diameter, with the laser energy at 170 nJ. The spot distance and tracking spacing were set at 4.5 *μ*m for the cap and lenticule and at 2.5 *μ*m for the side cuts. Side cut angles were set at 90°, incision position was set at 120°, and incision width was 2.5 mm. After completion of the laser firing, the cornea incision was opened with a Sinskey hook. Identification of the anterior and posterior surface edge of the lenticule was made, and the anterior surface of the lenticule was bluntly dissected with a Chansue dissector, followed by the posterior surface. The lenticule was then grasped and removed by a Tan DSAEK forceps (ASICO, Westmont, IL, USA). Finally, the corneal pocket was irrigated with 1.5 mL BSS using a 25-gauge Buratto's cannula for the SMILE with irrigation group, while the irrigation was not done for the SMILE without irrigation group. Immediately after the procedure, the eyes received topical tobramycin ointment once (Alcon, Fort Worth, TX, USA).

### 2.4. Clinical Evaluation

Rabbits were subjected to examination by slit lamp biomicroscopy (Nikon FS-3V; Nikon), Fourier-domain anterior segment optical coherence tomography (ASOCT; RTVue; Optovue, Inc, Fremont, CA), and in vivo confocal microscopy (IVCM; HRT3; Heidelberg Engineering GmbH, Heidelberg, Germany) preoperatively, immediately after surgery and at 4, 8, and 24 hours postoperatively. For ASOCT evaluation, the examiner adjusted the system to position the center of the treatment zone (6.5 mm laser ablated optical zone in the post-LASIK eyes and 6.5 mm lenticule zone in the post-SMILE eyes) at the center of the ASOCT image in order to maximize centration. Seventeen line raster images with an 8 mm scan length were taken for each eye at each time point. For each image, central corneal thickness and anterior lamellar thickness were measured in each raster line except at the measurement taken prior to the procedure, where only the total corneal thickness was measured. The posterior lamellar thickness was derived by subtracting the anterior lamellar thickness from the total corneal thickness. The average value of the 17 raster image measurements was calculated and used for further statistical analysis. For IVCM evaluation, Carbomer gel (Vidisic; Mann Pharma, Berlin, Germany) was applied on the confocal lens as the immersion fluid. The central treatment zone was examined with a minimum of three *z*-axis scans, consisting of the entire corneal thickness. For each eye, three micrographs of the cap or flap interface and of 10 *μ*m anterior and posterior to the interface were selected. These nine scans of each eye were further analyzed by semiquantifying the mean gray value of reflectivity using Image J (http://imagej.nih.gov/ij/; provided in the public domain by the National Institutes of Health, Bethesda, MD, USA), as described previously [18], and then the average value was calculated for statistical analysis.

### 2.5. Histology Analysis

At 24 hours postoperatively, the rabbits were euthanized under anesthesia by intracardiac injection of overdosed sodium pentobarbitone (Jurox, Rutherford, Australia). The eyes were enucleated and the corneas were excised. The samples were fixed in neutral 4% buffered paraformaldehyde (Sigma-Aldrich, Singapore), dehydrated, cleared, embedded in paraffin, and then cut in 7 *μ*m sections. The sections were air-dried for 10 minutes and rehydrated with 95% ethanol for 5 minutes. The slides were then washed prior to hematoxylin staining for 2.5 minutes followed by treatment with Scott's tap water for 5 minutes. They were then stained with Eosin for 2 minutes followed by washing in tap water. Series of dehydration with 95% and 100% ethanol were carried out for 5 minutes each. The sections were mounted after two changes of xylene for 2 minutes each and examined using Axioplan, Zeiss Light Microscope (Carl Zeiss MicroImaging), under bright field mode.

### 2.6. Statistical Analysis

All data were expressed as mean ± standard deviation (SD). Statistical comparisons among three groups were performed using Kruskal–Wallis test with Dunn post hoc tests. The percentage of change in the total corneal thickness and anterior and posterior lamellar thickness was calculated by using the value of preoperative total corneal thickness and by assuming that the postoperative anterior lamellar thickness was 110 *μ*m for the post-LASIK eyes and 120 *μ*m for the post-SMILE eyes. Statistical analyses were performed using STATA software (version 13, STATACrop, College Station, TX). *P* values less than 0.05 were considered statistically significant.

## 3. Results

### 3.1. Slit Lamp Evaluation

During the follow-up period of 24 hours, all the corneas either in the FS-LASIK or SMILE group (either with irrigation or without irrigation) remained clear, and no corneal edema or opacification was observed. All the flap incision wounds in the post-FS-LASIK eyes and small peripheral incision in the post-SMILE eyes were intact, without wound dehiscence or wound tears.

### 3.2. Corneal Thickness Evaluated by ASOCT

**Figure 1:**
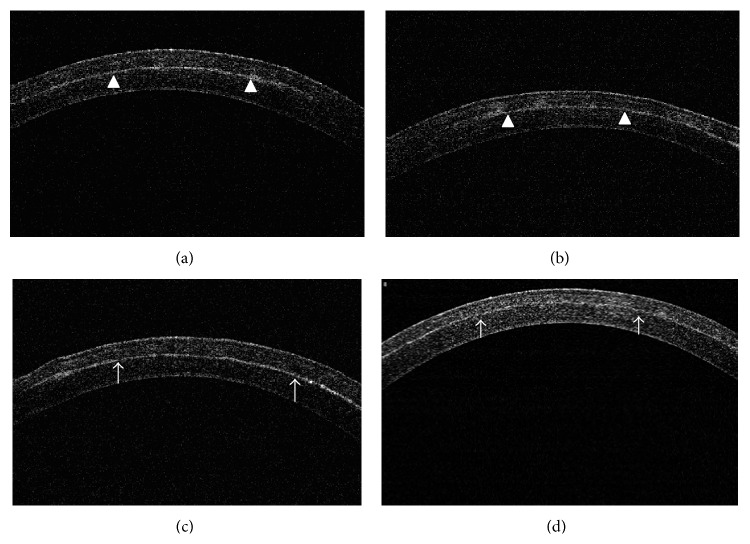
Representative raster line ASOCT images at 24 hours postoperatively for eyes which underwent (a) SMILE with irrigation, (b) SMILE without irrigation, (c) LASIK with irrigation, and (d) LASIK without irrigation procedure. An increase in the reflectivity could be seen at the cap interface in the post-SMILE eyes (arrowheads) or at the flap interface in the post-FS-LASIK eyes (arrow), and the total corneal thickness and anterior lamellar thickness were accordingly measured. The posterior lamellar thickness was then calculated by subtracting the anterior lamellar thickness from total corneal thickness.

**Figure 2:**
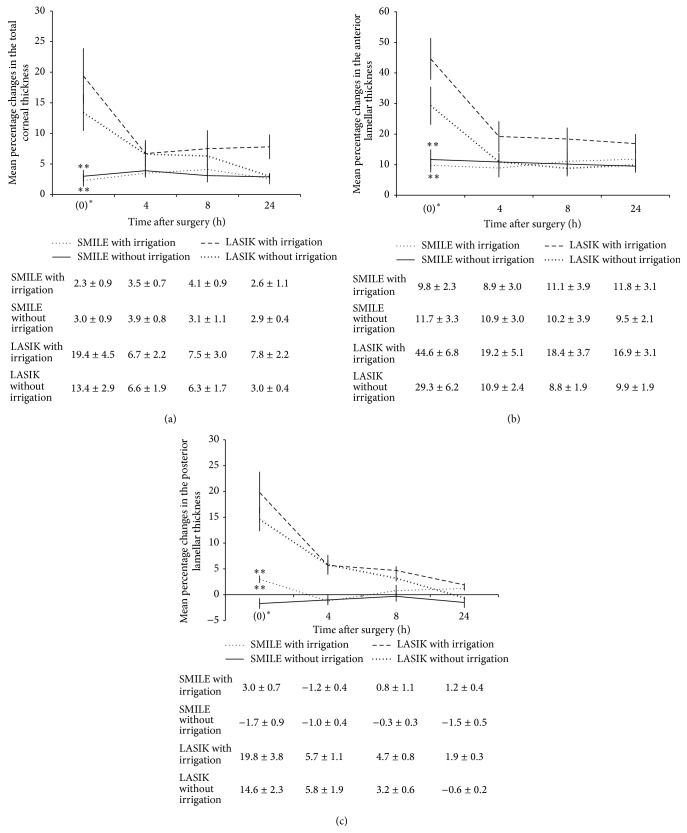
Line graphs showing the mean percentage changes in the (a) total corneal thickness, (b) anterior lamellar thickness, and (c) posterior lamellar thickness at different time points for different groups. 0 hr^*∗*^ indicates immediately after surgery. Error bars represent standard deviations (SD). The mean ± SD values for different groups at different time points were shown (%). The LASIK group had a significantly greater increase in the total corneal thickness and anterior lamellar and posterior lamellar thickness compared to the SMILE with irrigation and without irrigation groups immediately after surgery. The *P* level indicates the significance level when comparing the LASIK group (with irrigation and without irrigation) with other groups: ^*∗∗∗*^ *P* < 0.001 and ^*∗∗*^ *P* < 0.01.

On ASOCT examination, an increase in the reflectivity could be seen at the flap interface in the post-FS-LASIK eyes or cap interface in the post-SMILE eyes (either with or without irrigation) at all the examination time points (Figure 1). The preoperative corneal thickness was 377.8 ± 14.4 *μ*m, 377.5 ± 8.1 *μ*m, 382.0 ± 2.6 *μ*m, and 377.0 ± 15.5 *μ*m for the FS-LASIK with irrigation, FS-LASIK without irrigation, SMILE with irrigation, and SMILE without irrigation groups, respectively (*P* = 0.883). After surgery, the mean percentage changes in the total corneal thickness, anterior lamellar thickness, and posterior lamellar thickness, at different time points for the four groups, are shown in Figure 2.

Immediately after surgery, the post-FS-LASIK eyes, either with or without irrigation, had a significantly greater increase in the total corneal thickness than the post-SMILE eyes (either with or without irrigation; all *P* < 0.01). Thereafter, the amplitude of thickness change in the post-FS-LASIK eyes, either with irrigation or without irrigation, gradually decreased. There was no significant difference in the total corneal thickness, anterior lamellar thickness, or posterior lamellar thickness between FS-LASIK with irrigation and FS-LASIK without irrigation groups or SMILE with irrigation and SMILE without irrigation groups. When comparing the SMILE with irrigation to SMILE without irrigation groups, both groups had an increase in the total thickness and anterior lamellar thickness after the surgery. In the SMILE with irrigation group, the total thickness increased by 2.3 ± 0.1%, 3.5 ± 0.7%, 4.1 ± 0.9%, and 2.6 ± 1.1%, immediately after surgery and at 4, 8, and 24 hours postoperatively, whereas the total thickness increased by 3.0 ± 1.1%, 3.9 ± 0.8%, 3.1 ± 1.1%, and 2.9 ± 0.4% immediately after surgery and at 4, 8, and 24 hours postoperatively in the SMILE without irrigation group (*P* = 0.838, *P* = 0.465, *P* = 0.844, and *P* = 0.267, resp.). When looking at the anterior cap thickness, it was also comparable in both groups at all the examination time points. In the SMILE with irrigation group, the anterior lamellar thickness increased by 9.8 ± 2.3%, 8.9 ± 3.0%, 11.1 ± 3.8%, and 11.8 ± 3.1% immediately after surgery and at 4, 8, and 24 hours postoperatively, whereas the SMILE without irrigation group showed increase by 11.7 ± 3.3%, 10.9 ± 2.8%, 10.2 ± 4.0%, and 9.5 ± 2.1% immediately after surgery and at 4, 8, and 24 hours postoperatively (*P* = 0.256, *P* = 0.391, *P* = 0.267, and *P* = 0.632, resp.).

### 3.3. In Vivo Confocal Micrographs Analysis

**Figure 3:**
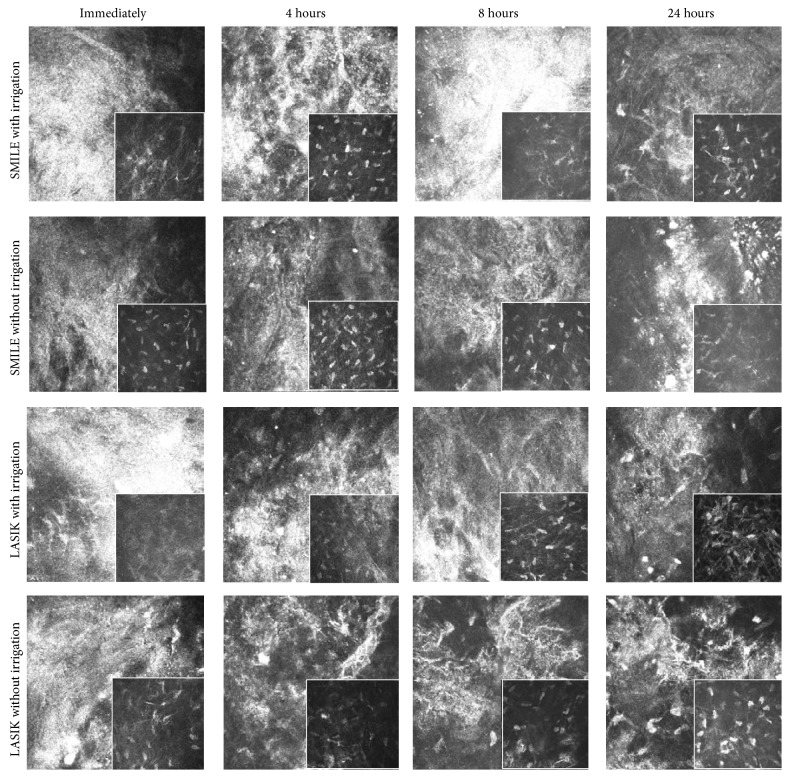
Representative in vivo confocal images at the extracted lenticule plane (SMILE with and without irrigation groups) and at the flap interface (LASIK with and without irrigation groups) at different time points. These interface layers were acellular and were characterized by light-scattering particles. Inset images showed the plane adjacent to the extracted lenticule plane or flap interface. Highly reflective keratocytes were observed in these planes.

**Figure 4:**
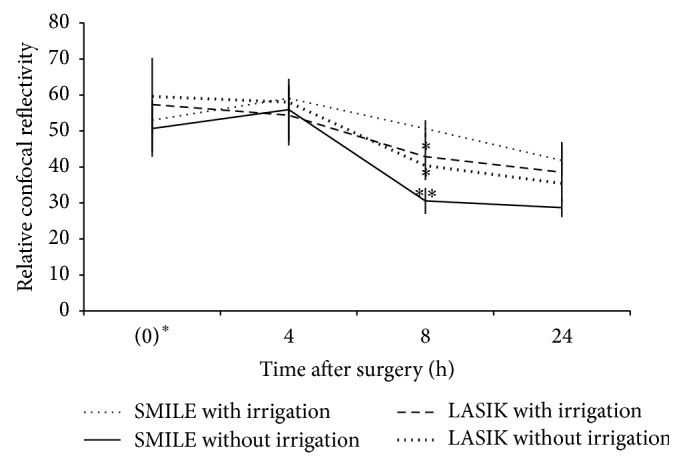
Line graph showing the relative intensity of reflectivity for different groups at different time points. At postoperative 8 hours, the SMILE with irrigation group had significantly higher reflectivity than the other three groups. 0 hr^*∗*^ indicates immediately after the surgery. The *P* level indicates the significance level when comparing the SMILE with irrigation group with other groups: ^*∗*^ *P* < 0.05 and ^*∗∗*^ *P* < 0.01. Error bars represent SD.

Highly reflective keratocytes were observed in the stromal layer directly anterior and posterior to the excimer laser ablation plane in the post-LASIK eyes or to the extracted lenticule plane in the post-SMILE eyes. The keratocytes were observed in similar density in all corneas. The flap interface and extracted lenticule plane were acellular and characterized by light-scattering particles (Figure 3). Semiquantitative analysis of the reflectivity was performed. The reflectivity in the four groups was comparable within the first 4 hours (*P* = 0.693 and *P* = 0.745, resp., immediately after surgery and 4 hours postoperatively). Immediately after surgery, post-FS-LASIK eyes (with irrigation or without irrigation) had higher reflectivity than post-SMILE eyes (with irrigation or without irrigation), although the difference was not statistically significant. At postoperative 8 hours, the eyes that underwent SMILE with irrigation had significantly higher reflectivity than those which underwent FS-LASIK with irrigation, FS-LASIK without irrigation, and SMILE without irrigation (*P* = 0.047, *P* = 0.041, and *P* = 0.005, resp.). The reflectivity decreased gradually thereafter. At postoperative 24 hours, the reflectivity decreased by 33.1%, 40.6%, 42.7%, and 21.4%, respectively, in the FS-LASIK with irrigation, FS-LASIK without irrigation, SMILE without irrigation, and SMILE with irrigation groups, when compared to that immediately after surgery (*P* = 0.019, *P* = 0.046, and *P* = 0.116, resp.). At 24 hours, the reflectivity difference among different groups was not statistically significant (*P* = 0.083; Figure 4).

### 3.4. Histological Analysis

**Figure 5:**
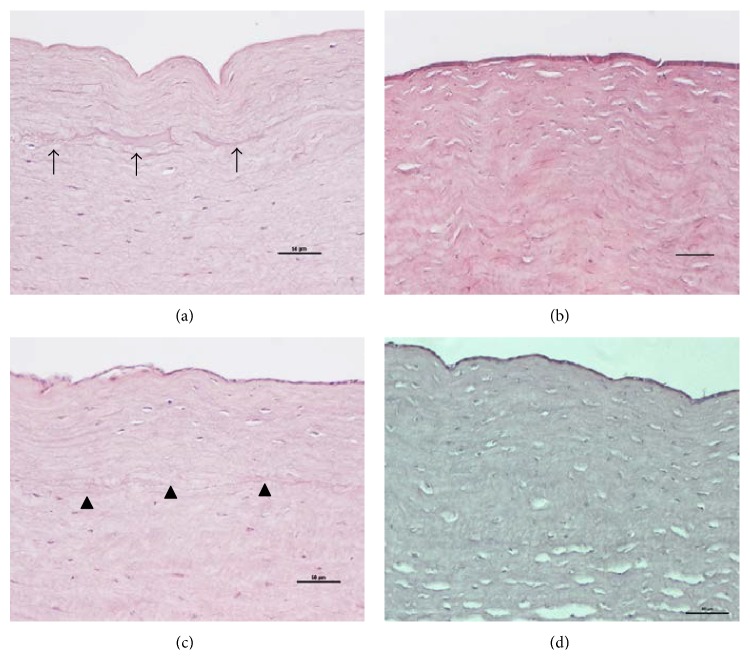
Histological section with H&E staining for the central corneas of the (a) SMILE with irrigation, (b) SMILE without irrigation, (c) LASIK with irrigation, and (d) LASIK without irrigation groups at 24 hours. In the SMILE with irrigation group, undulated but undisrupted stromal collagen bundles and apparent fluid pockets along the extracted lenticule plane were observed (arrows). Small fluid retention pockets were also observed at the interface in the post-LASIK eyes but less prominent (arrowheads). Original magnification: 200x. Scale bar: 50 *μ*m.

At 24 hours after surgery, the central corneas of the four different groups had normal epithelial and endothelial cells, with comparable total corneal thickness. The central corneas in the SMILE with irrigation group had noticeably undulated but undisrupted stromal collagen bundles in the anterior and posterior stroma, compared to those in the SMILE without irrigation group. Undulated stromal collagen was also observed in the central corneas of post-FS-LASIK eyes but less abundant (Figure 5). The extracted lenticule plane in the post-SMILE eyes and flap interface in the post-FS-LASIK eyes could be delineated. Particularly, small pockets with fluid retention were observed along the extracted lenticule plane in the majority of corneas of SMILE with irrigation group (5/8 eyes). Fluid pockets were also noted in the post-FS-LASIK with irrigation corneas but less prominent. In all corneas, there were no apparent infiltrated inflammatory cells or obvious fibrotic tissue formation.

## 4. Discussion

During the last 2 years, SMILE has become clinically available in Europe and Asia as an alternative to LASIK for the correction of myopia or astigmatism [2]. In the present study, we demonstrated that flushing the corneal pocket with BSS in the SMILE procedure did not result in significant changes in corneal thickness. The eyes with irrigation had a transient increase in the interface reflectivity as demonstrated in IVCM, and the histological analysis revealed undulated but undisrupted collagen with fluid pockets after irrigation.

The corneal thickness was evaluated with RTVue ASOCT. This device has an optical resolution of 5 *μ*m and is shown to provide consistent measurements for flap or cap thickness [19, 20]. All study eyes had a postoperative increase in the corneal thickness during the study period of 24 hours, and the changes in the thickness mainly occurred in the anterior lamellae (flap or cap). The FS-LASIK group, either with or without irrigation, had a significantly greater increase in the total corneal thickness and anterior lamellar and posterior lamellar thickness, measured immediately after surgery, compared to the other two groups. This may result from the greater flap manipulation from the complete flap lifting in FS-LASIK. Similarly, in our published study, we have also found a larger amount of flap swelling after femtosecond lenticule extraction (FLEx) than after SMILE due to the greater flap manipulation [20]. Thereafter, the total corneal thickness and anterior and posterior lamellar thickness in the post-FS-LASIK eyes gradually decreased with time, with a thickness change of 16.9% in the flap at 24 hours postoperatively in the FS-LASIK with irrigation group. This was in agreement with our previous clinical study, reporting a thickness change of approximately 11% in the flap following FS-LASIK [19]. In comparison, the total corneal thickness and anterior and posterior lamellar thickness in the SMILE eyes, either in the irrigated or nonirrigated group, appeared stable after the surgery (Figure 2). The SMILE with irrigation eyes did not have significantly thicker cornea compared with those without irrigation throughout the study period, which means there was minimal absorption of the irrigating fluid, and the corneal edema came from surgical manipulation of the lenticule extraction, not from the fluid.

In vivo confocal microscopy has been used to evaluate corneal stromal reaction and keratocyte activation [21–23]. In all corneas, we visualized the activated keratocytes by recognizing their cell bodies and processes at the anterior and posterior lenticule planes [23]. Acellular zone with light-scattering particles was observed at the flap interface and extracted lenticule plane. The presence of acellular zone was due to keratocyte apoptosis and the formation of hypocellular primitive stromal scar [24]. Interface light-scattering particles have been observed after microkeratome-LASIK [25, 26], FS-LASIK [27, 28], FLEx [21], and SMILE [27]. The nature of these reflective particles has been widely speculated upon. Various suggestions are metal pieces or plastic particles from the instrument, ocular surface debris such as lipid products or implanted corneal epithelium, synthetic material such as cotton fibers or sponge particles from absorbing substances used during surgery, powder from surgical gloves, or inflammatory cells in the wound [24]. Dawson et al. found that these reflective particles consist primarily of organic cellular constituents in the histologic studies, and some of which were transient in nature [24]. Immediately after surgery, the FS-LASIK eyes, either with irrigation or without irrigation, had higher reflectivity than the SMILE eyes (both with irrigation and without irrigation groups). It may be associated with greater tissue injury in a FS-LASIK than a SMILE procedure, as excimer laser causes more tissue injury than femtosecond laser [21, 29]. The reflectivity in the post-FS-LASIK eyes gradually declined with time. At 4 hours postoperatively, the post-SMILE eyes had a transient increase in the reflectivity. The reasons for that were unclear, but we thought it might be because the released inflammatory cytokines, peaking within few hours after surgery [30], stayed in the pocket more and longer than under the flap, as the former is a relatively “closed” system. At 8 hours postoperatively, significantly higher interface reflectivity was observed in the SMILE with irrigation group compared to the SMILE without irrigation group. It was also significantly higher than that in the FS-LASIK with irrigation group, although both had BSS irrigation during the procedure. On the next day of surgery, however, no difference was seen in the reflectivity among four groups, although the SMILE with irrigation group had the least reduction in interface reflectivity. The eyes with irrigation, either in the LASIK or SMILE group, had less and slower reduction in the reflectivity, compared to those without irrigation.

On histological examinations, fluid pockets were observed along the extracted lenticule plane in the SMILE with irrigation group on the next day of the surgery. We postulate that this subtle fluid layer may affect the immediate contrast sensitivity and result in blurring of vision in the very early postoperative period. However, attesting whether our notion of irrigation is associated with this phenomenon requires a further clinical study. Contrast sensitivity is thought to be a factor affecting the subjective quality of vision [31]. Ganesh and Gupta have reported that at day 1 postoperatively, the contrast sensitivity was better in femtosecond-LASIK patients than in SMILE patients. However, by day 15, the contrast sensitivity improved in the SMILE group, and the difference between the SMILE and LASIK groups was no longer significant [8]. Our histological data may explain these clinical observations that the transient intrastromal fluid retention coming from the irrigation may affect the visual quality in the early SMILE postoperative period. Our presupposition that the irrigation fluid in the FS-LASIK eyes was more likely to escape from the intrastromal space was also confirmed by the histological analysis and IVCM. The amount and extent of fluid pockets in the interface were less apparent than those in the SMILE with irrigation eyes. The less obvious fluid pockets in the FS-LASIK with irrigation eyes shown on the histological analysis also supported our thoughts that the flap swelling of FS-LASIK eyes came from surgical manipulation.

## 5. Conclusions

In this study, we reported the corneal lamellar thickness changes, stromal response by IVCM, and histological changes for the pocket irrigation step in SMILE and compared them to those in FS-LASIK, using a rabbit model. The flushing step led to a transient increase in the interface reaction, but the activity subsided on the following day after surgery. It did not result in significant corneal thickness change. The presence of interface fluid was confirmed by the identification of fluid pockets shown on the histological section. This might contribute to the delayed immediate postoperative visual quality following SMILE and further clinical studies are required. However, in view of the benefits of washing, that is, removal of inflammatory debris, we would still advocate the irrigation step when performing SMILE but would also combine it with gentle massaging of the anterior cap towards the incision to reduce the amount of interface fluid.
